# The CKD-OSTEO Score: A Chronic Kidney Disease-specific Tool for Osteoporosis Risk Stratification

**DOI:** 10.17925/EE.2026.22.1.2

**Published:** 2026-02-11

**Authors:** Sourabh Sharma, Sanjay Kalra, Nitin Kapoor, Lakshmi Nagendra, Ashish Jaiman, Sree Bhushan Raju

**Affiliations:** 1. Department of Nephrology, Vardhman Mahavir Medical College and Safdarjung Hospital, New Delhi, India; 2. Department of Endocrinology, Bharti Hospital, Karnal, India; 3. University Centre for Research and Development, Chandigarh University, Mohali, India; 4. Department of Endocrinology, Diabetes and Metabolism, Christian Medical College, Vellore, India; 5. Non Communicable Disease Unit, Baker Heart and Diabetes Institute, Melbourne, Victoria, Australia; 6. Department of Endocrinology, JSS Medical College, JSS Academy of Higher Education and Research, Mysore, Karnataka, India; 7. Central Institute of Orthopaedics, Vardhman Mahavir Medical College and Safdarjung Hospital, New Delhi, India; 8. Department of Nephrology, Nizams Institute of Medical Sciences, Hyderabad, Telangana, India

**Keywords:** Bisphosphonates, chronic kidney disease, fibroblast growth factors, intact parathormone, Klotho, metabolic bone disease, osteoporosis, parathyroid hormone

## Abstract

Osteoporosis in patients with chronic kidney disease (CKD) frequently goes undiagnosed and is not treated adequately, largely due to its intricate pathophysiology and the lack of risk stratification tools specific to CKD. Standard tools used for the general populace, such as the Fracture Risk Assessment Tool (FRAX) score and bone mineral density-based thresholds, fail to accurately represent the unique biochemical and structural bone alterations that take place in CKD stages IIIb–VD. We propose the Chronic Kidney Disease Osteoporosis Stratification Tool for Evaluation and Optimization (CKD-OSTEO) score, an innovative multidimensional clinical tool intended to evaluate the risk of osteoporosis, direct diagnostic assessments and inform suitable treatment strategies for patients with CKD. This score integrates conventional fracture risk factors with CKD-specific components, such as dialysis vintage, intact parathormone levels, serum Klotho levels and T-scores. We explain the pathophysiological rationale for each element, categorize patients into various management levels and provide customized recommendations for starting and selecting anti-resorptive therapy. Emphasis is placed on the growing importance of Klotho deficiency as a marker of bone–vascular ageing. With this article, we advocate for the regular risk stratification of patients with CKD to enhance fracture prevention, tailor treatment strategies and incorporate new biomarkers like Klotho into practice.

The significance of osteoporosis and fragility fractures in individuals with chronic kidney disease (CKD) is becoming increasingly recognized as a major contributor to disability, hospital admissions and mortality. In CKD, bone fragility is worsened by the complex interactions of metabolic bone disorders, uraemic toxins, systemic inflammation, sarcopenia and changes in the bone–vascular axis.^1,2^ Recent studies indicate that in women diagnosed with osteoporosis, more than 60% were found to have an estimated glomerular filtration rate (eGFR) of 30–59 mL/min (CKD stage III), and 23% had an eGFR of 15–29 mL/min (CKD stage IV).^1^ In the geriatric population diagnosed with osteoporosis, around one-fourth of those aged 70–79 years, and more than half of those over 80 years, had an eGFR <35 mL/min.^1^ The current approach to managing osteoporosis in CKD lacks coherence and consistency, primarily due to the absence of a unified risk stratification system. Current assessment tools, such as FRAX, often fail to accurately estimate fracture risk in CKD and usually underestimate the risk, as they overlook crucial factors such as glomerular filtration rate, dialysis vintage, parathormone (PTH) levels, bone-turnover markers and emerging indicators such as Klotho.^3,4^ Furthermore, past concerns about inducing adynamic bone disease have led to hesitance in using bisphosphonates and denosumab in advanced CKD stages.^5,6^ With growing evidence supporting personalized treatment in CKD-metabolic bone diseases (CKD-MBDs) and the availability of Klotho tests, there is an urgent need for a customized scoring system for CKD to improve decision-making. We introduce the Chronic Kidney Disease Osteoporosis Stratification Tool for Evaluation and Optimization (CKD-OSTEO) score, a nine-point multidimensional evaluation tool that assesses the risk of osteoporotic fractures and offers tiered, evidence-based recommendations for further evaluation and treatment.

## Chronic Kidney Disease Osteoporosis Stratification Tool for Evaluation and Optimization score components

The score incorporates nine parameters (*Table 1*), each selected for its pathophysiological significance and predictive potential in CKD-related bone fragility. The maximum score is 18. The components can be divided into clinical and investigatory categories, as enumerated below:

### Clinical components

**Age (0–2 points):** Age is a universal risk factor for osteoporosis. Older individuals with CKD have a higher baseline fracture risk, compounded by CKD-related skeletal changes.^7,8^ The two-point weight reflects the strong, graded association in meta-analyses by Iseri et al. (Swedish Renal Registry).^9^

**Table 1:** Chronic Kidney Disease Osteoporosis Stratification Tool for Evaluation and Optimization score components

	Category	Parameter	Score*	Stratification
**Clinical**	Age	0–2	<50 years: 0 50–64 years: 1 ≥65 years: 2
	Gender	0–2	Male: 0 Reproductive age female: 1 Post-menopausal female: 2
	CKD stage	0–3	Stage IIIb: 0 Stage IV: 1 Stage V (non-dialysis): 2 Stage VD (dialysis): 3
	Dialysis vintage	0–2	<1 year: 0 1–3 years: 1 >3 years: 2
	Previous fragility fracture	0–2	None: 0 One fracture: 1 Multiple fractures: 2
	Risk-enhancing comorbidity (diabetes/RhA/smoking)	0–1	Absent: 0 Any one or more present: 1
	Conmeds (corticosteroids/PPI/warfarin/CNI)	0–1	Absent: 0 Present: 1
	Frail by index	0–1	Absent: 0 Present: 1
**Expanded CKD-OSTEO**			
**Investigations**	iPTH level (pg/mL)	0–1	100–600: 0 <100 or >600: 1
	T-score (DEXA)	0–1	>-1.0: 0 -1.0 to -2.5: 1*
	Serum Klotho level (as per availability)	0–2	Normal or not measured: 0 Low: 2
**To consider**	**Bone-turnover markers (e.g. b-ALP, TRACP-5b)**		

*Scores 1–3 low risk; 4–6 moderate risk; ≥7 high risk for osteoporosis.*

*Osteopenia is a risk factor for osteoporosis.

*b-ALP = bone-specific alkaline phosphatase; CKD = chronic kidney disease; CNI = calcineurin inhibitor; Conmeds = concomitant medications; DEXA = dual-energy X-ray absorptiometry; iPTH = intact parathormone levels; PPI = proton pump inhibitor; RhA = rheumatoid arthritis; TRACP-5b = Tartrate-Resistant Acid Phosphatase Isoform 5b.*

**Gender (0–2 points):** As in the general population, osteoporosis is more common in females, and it is even more prevalent in post-menopausal females with CKD, justifying the two-point weight.^7,10^

**CKD stage (0–3 points):** Higher stage reflects progressive reductions in eGFR and associated abnormalities in bone turnover and mineral metabolism (CKD-MBD).^7,11^ A three-point scale reflects linear deterioration with eGFR decline and CKD progression.^1^

**History of fragility fracture (0–2 points):** Past low-impact fragility fractures are highly predictive of future fractures.^1,2^ As per the meta-analysis by Kanis et al., fracture risk gets doubled in cases with a prior history of fracture.^12^

**Risk-enhancing comorbidity (0–1 point):** Each comorbidity, including diabetes, smoking and rheumatoid arthritis, modestly increases bone fragility via microvascular or inflammatory pathways.^13^

**Concomitant medications (0–1 point):** Includes chronic (more than 1 month) use of corticosteroids, proton pump inhibitors, warfarin or calcineurin inhibitors.^14^ This binary inclusion aligns with secondary osteoporosis risk frameworks such as FRAX score.^3,4^

**Frail by index (0–1 point):** Frailty due to non-ambulation is a known independent risk factor for osteoporosis.^15^

**Dialysis vintage (0–2 points):** Longer dialysis duration increases the risk of low-turnover bone disease and vascular calcification.^7^

### Investigatory components

**PTH level (0–1 point):** Both low and excessively high PTH levels are associated with impaired bone remodelling, justifying the binary inclusion.^4,5^

**T-score from dual-energy X-ray absorptiometry (DEXA; 0–1 point):** A T-score <-2.5 indicates osteoporosis; however, DEXA alone may underestimate microarchitectural damage in CKD. DEXA indicative of osteopenia (-1 to -2.5) is a risk factor for osteoporosis and has been weighted 1 to maintain additive balance with clinical factors.^3,4^

**Serum Klotho level (0–1 point):** Low Klotho levels correlate with poor bone quality, sarcopenia and vascular calcification.^16^ Given its causal and integrative influence, it has been assigned two points.

### To consider

**Bone-turnover markers (e.g. bone-specific alkaline phosphatase, tartrate-resistant acid phosphatase isoform 5b):** Not scored but used to refine decision-making.^17^ Their interpretation in CKD is assay-dependent and confounded by reduced renal clearance and variable reference ranges across CKD stages.

The CKD-OSTEO score can facilitate an individualized, CKD-specific osteoporosis management. The various advantages that this score yields over existing tools are as follows:

- This score is CKD-adapted and includes dialysis vintage and Klotho levels.
- It is applicable across CKD stages (stages IIIb–VD).
- It facilitates targeted initiation of anti-osteoporosis therapy.
- It integrates both traditional and emerging biomarkers.

*Table 2* depicts the comparative context between CKD-OSTEO and existing osteoporosis risk tools.^18^ Unlike the FRAX score, which is validated for the general population, the CKD-OSTEO score incorporates kidney-specific risk factors within an accessible framework. This score aligns with the Kidney Disease: Improving Global Outcomes (KDIGO) 2024 draft, which emphasizes integrated risk assessment over isolated measurements, and presents a clinically actionable format suitable for screening osteoporosis in patients with CKD.^18^

## Practical integration

In the nephrology out-patient department, the CKD-OSTEO score should be calculated annually in all patients with stages IIIb–VD. The score can be documented in CKD-MBD flow sheets, and a score ≥7 should be indicated as a red flag for urgent intervention.

**Table 2:** Comparative context between Chronic Kidney Disease Osteoporosis Stratification Tool for Evaluation and Optimization score and existing osteoporosis risk tools^18^

Domain	CKD-OSTEO score	FRAX tool	KDIGO CKD-MBD guidelines^18^
**Primary purpose**	Stratifies osteoporosis and fragility risk in CKD stages IIIb–VD	Estimates 10-year fracture risk in general population	Provides framework for managing CKD-MBD
**Population**	Developed specifically for patients with CKD and dialysis (validation needed)	Validated in general population, may underestimate risk in CKD	Guidelines, not a scoring system
**Included parameters**	Clinical (age, sex, comorbidities, dialysis vintage), biochemical (PTH, Klotho) and structural (DEXA)	Age, sex, BMI, fracture history, steroids, smoking	Recommends considering iPTH, ALP, bone biopsy, imaging
**CKD-specific markers**	Includes CKD stage, dialysis vintage, iPTH extremes, Klotho	Not included	Mentions but not quantified
**Bone-turnover integration**	Option to include while managing high-risk cases	Not included	Advises measurement but no scoring framework
**Clinical usability**	Point-based bedside scoring Adaptable for risk stratification and intervention planning	Online calculator, population-specific thresholds	No numeric risk
**Clinical advantage**	Provides a CKD-adapted, integrative and pragmatic tool for routine use	General population baseline comparator	Not a quantitative model

*ALP = alkaline phosphatase; BMI = body mass index; CKD = chronic kidney disease; CKD-MBD = chronic kidney disease-mineral and bone disorder; CKD-OSTEO = Chronic Kidney Disease Osteoporosis Stratification Tool for Evaluation and Optimization; DEXA = dual-energy X-ray absorptiometry; FRAX = Fracture Risk Assessment Tool; iPTH = intact parathormone levels; KDIGO = Kidney Disease: Improving Global Outcomes; PTH = parathormone.*

## Interpretation and management

The CKD-OSTEO score guides not only fracture risk stratification but also evaluation and treatment decisions. *Table 3* depicts a clinical algorithm based on the CKD-OSTEO score. DEXA is a confirmatory test and needs to be performed in any case with a risk of osteoporosis.

## Pharmacotherapy

Bisphosphonates are indicated in osteoporosis in CKD stages IIIb–IV with a T-score <-2.5 or a fragility fracture; CKD stage VND or VD with high intact PTH level (iPTH; >600 pg/mL), and a T-score <-2.5 and/or a fragility fracture; or a CKD-OSTEO score ≥7 with no contraindications. Bisphosphonates are contraindicated in suspected low-turnover bone disease unless confirmed by biopsy. If bone-turnover markers (low alkaline phosphatase, low PTH) suggest adynamic bone disease, antiresorptive therapy should be deferred.^6,7,17^

The choice of bisphosphonate must consider renal clearance, bone-turnover status and dialysis status. In CKD stages III–IV, alendronate or risedronate can be used cautiously with monitoring. In CKD stage VD, pamidronate or low-dose intravenous (IV) zoledronic acid (up to 4 mg yearly) may be considered cautiously in high-turnover states with close PTH monitoring.^6^

Denosumab is preferred in advanced CKD and dialysis-dependent patients due to a lack of renal clearance and strong efficacy, but hypocalcaemia is a significant risk, particularly in those with low bone-turnover states or adynamic bone disease.^19^

Romosozumab is a humanized monoclonal antibody that targets and inhibits sclerostin, leading to the activation of the Wnt pathway, which promotes osteoblast survival, proliferation and bone formation while simultaneously reducing bone resorption. CKD is associated with elevated serum sclerostin levels, which may contribute to impaired bone formation by further inhibiting the Wnt signalling pathway. Thus, romosozumab’s mechanism of action directly addresses a key pathological feature in CKD-related bone disease.^20^

Romosozumab has been shown to significantly increase bone mineral density (BMD) and reduce fracture risk in postmenopausal women with osteoporosis, including those with mild-to-moderate CKD (eGFR down to 30 mL/min). *Post hoc* analyses from large trials, such as FRAME (FRacture Study in Postmenopausal WoMen with OstEoporosis; C linicalTrials. gov identifier: NCT01575834), indicate that the efficacy and safety profile of romosozumab remains consistent across various stages of kidney function, at least down to moderate CKD. However, data on patients with severe renal impairment remain limited.^20^

**Table 3:** Clinical algorithm based on Chronic Kidney Disease Osteoporosis Stratification Tool for Evaluation and Optimization score

Score	Workup	Treatment
**1–3 (low risk)**	DEXA + baseline iPTH	Lifestyle interventions (exercise) Nutrition (vitamin D repletion, phosphate control) Fall prevention
**4–6 (moderate risk)**	Detailed biochemical evaluation (iPTH, calcium, phosphate, 25-OH vitamin D, ALP) Imaging (DEXA ± spine X-ray) Serum Klotho level	Non-pharmacological measures as above + treat with anti-osteoporosis therapy if fragility fracture/T-score <-2.5 with altered iPTH
**≥7 (high risk)**	Further imaging (BMD + TBS) Evaluate bone turnover Bone biopsy (if available) if turnover status is uncertain Vascular calcification screening	Non-pharmacological therapy + pharmacologic therapy regardless of eGFR and T-score (bisphosphonates/denosumab/anabolic therapy as per CKD stage and bone turnover) Ensure corrected calcium and iPTH prior to initiation

*ALP = alkaline phosphatase; BMD = bone mineral density; CKD = chronic kidney disease; DEXA = dual-energy X-ray absorptiometry; DXA = dual-energy X-ray absorptiometry; eGFR = estimated glomerular filtration rate; iPTH = intact parathormone levels; 25-OH = 25-hydroxy; TBS = trabecular bone score (assessed for bone quality – calculated from the grey-level texture of DXA images, using a modified approach of the known experimental variogram).*

## Anabolic therapy

Anabolic therapy using PTH analogues such as teriparatide and abaloparatide is effective and generally safe for postmenopausal women with osteoporosis and CKD stages I–III, if they do not have high levels of endogenous PTH. In CKD stages IV–V, there is limited evidence indicating possible benefits, particularly for those with adynamic bone disease, although careful monitoring is necessary. Teriparatide could lead to hypercalcaemia and hyperuricaemia, but these effects typically do not result in serious clinical issues. The usual treatment duration is capped at 2 years, although the US Food and Drug Administration (FDA) has eased this limitation for patients considered high risk. Overall, tailored use in advanced CKD might be advantageous but must be approached with caution.^21^

## The role of Klotho in fracture prediction and systemic ageing

Klotho deficiency is increasingly being recognized as a significant factor in the premature ageing phenotype of CKD, which includes osteopenia, sarcopenia, vascular stiffness and cognitive decline. Klotho plays a crucial role in modulating fibroblast growth factor 23 (FGF-23) and phosphate metabolism, and its deficiency is independently associated with a higher risk of fractures, arterial calcification and decreased lifespan.^16^ Soluble Klotho assays, based on enzyme-linked immunosorbent assay (ELISA), are being adopted in Japan and Europe to enhance osteoporosis and frailty risk assessments in nephrology practices.^22^

## Limitations/opportunities

The score needs validation through both community-based and institution-based studies. The validation study could be done against prospectively ascertained incident fragility fractures, using radiographic confirmation as the primary clinical outcome, and cross-sectional BMD by DEXA and vertebral fracture assessment as secondary diagnostic outcomes. Validation should be event-driven, with an aim for at least 100 outcome events. In developing countries, soluble Klotho assays or bone-turnover markers are often difficult to obtain and can be expensive, with a lack of standardization. To maintain the applicability of the CKD-OSTEO score in these settings, Klotho has been included as an expanded parameter. In resource-limited settings, we suggest pragmatic alternatives (FGF-23 levels, iPTH or phosphate-retention indices) or research collaborations until reliable, validated Klotho assays become more widely accessible. However, there is a need for a risk calculator specifically adapted for CKD, and the CKD-OSTEO score may serve as a suitable scoring system. This score should be applied to all patients undergoing DEXA scans, which can ultimately help reduce bone morbidity. It is likely that India and other low-to middle-income countries will soon have access to affordable assays as part of standard CKD-MBD panels.

## Conclusion

The CKD-OSTEO score serves as a practical and pathophysiologically relevant clinical instrument for evaluating the risk of osteoporosis in CKD. It promotes early detection of individuals at high risk, encourages appropriate investigations and facilitates the initiation of safe therapies tailored to disease stage. Incorporating Klotho into clinical practice provides a significant prognostic and therapeutic indicator. We propose routine use of the CKD-OSTEO score in out-patients with CKD to help decrease the incidence of fractures and enhance musculoskeletal health outcomes.
